# Antony van Leeuwenhoek, Pioneer of the Microscopic World

**DOI:** 10.3201/eid3206.260531

**Published:** 2026-06

**Authors:** Robert Gaynes

**Affiliations:** Emory University School of Medicine, Atlanta, Georgia, USA

**Keywords:** bacteria, parasites, Giardia, Antony van Leeuwenhoek, microscopes, Vermeer, microbiology, the Netherlands

## Who is this person and what did he accomplish?

Here is a clue: He devised high-powered microscopes that extended our perception beyond the naked eye to the microbial world.

Who is he?

1. Robert Hooke

2. Jan Swammerdam

3. Thomas Molyneux

4. Antony van Leeuwenhoek

5. Nicolaas Hartsoeker

Decide first, then see the next page for the answer.

This is a painting of Antony van Leeuwenhoek (1632–1723), who is sometimes credited with inventing the microscope; he did not (Figure). However, he did markedly enhance it, devising a high-powered microscope that opened up the microscopic world. Others have seen deeper into this tiny domain, but van Leeuwenhoek was the first to witness it. He extended our perception beyond what could be seen with the naked eye to directly observe an entirely new universe.

**Figure:**
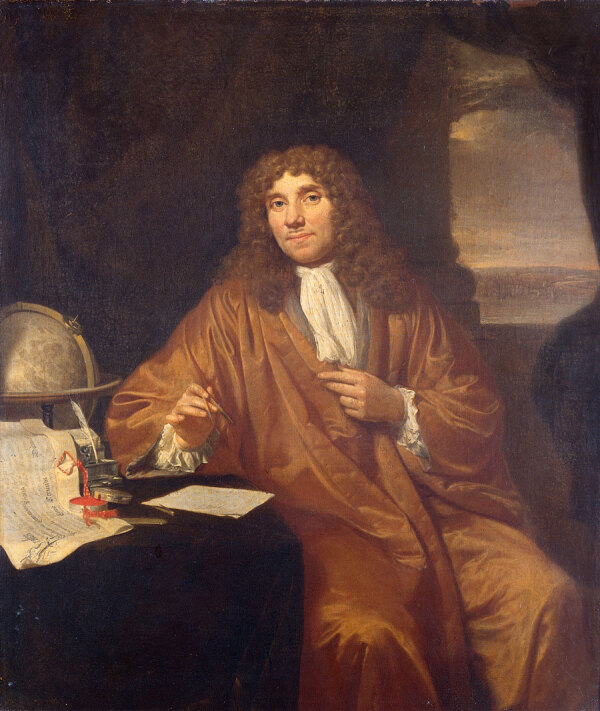
Antony van Leeuwenhoek (1632–1723). Painting by Jan Verkolje (1680–1686). Public domain image. Source: Wikimedia Commons.

Antony van Leeuwenhoek was born in 1632 in a small village in Holland named Delft. That was also the same town and year that Dutch painter Jan Vermeer entered the world. Their lives would take different paths but intermingle (*1*).

At age 16, van Leeuwenhoek went to Amsterdam and learned the drapery business. After about 6 years in Amsterdam, van Leeuwenhoek returned to Delft, where he began a reasonably successful drapery business. In 1660, he was appointed to a local government post that he held for 39 years. That position led van Leeuwenhoek to be appointed as official receiver of the estate of the painter Jan Vermeer when he died at age 43. Art historians have hypothesized that van Leeuwenhoek may have been the subject in 2 of Vermeer’s paintings—*The Geographer* and *The Astronomer*.

The salary from his local government post, along with an inheritance after his mother’s death in 1664, enabled van Leeuwenhoek to pursue interests outside his drapery business. Notably, van Leeuwenhoek began crafting lenses that he placed between 2 metal plates. With this handheld microscope, van Leeuwenhoek began to examine all sorts of objects. So-called microscopes up to that point were essentially magnifying glasses, capable of enlarging 20 times. van Leeuwenhoek created single-lens microscopes that could magnify 270 times and some as much as 500 times, a magnification far greater than that of any previous microscope. His skill at producing high-quality lenses for microscopes eventually caught the attention of the secretary of the British Royal Society, Henry Oldenburg, who was impressed. For the next 50 years, until his death, van Leeuwenhoek sent hundreds of letters describing his investigations to the Royal Society.

van Leeuwenhoek’s observations on single-celled organisms began in 1674 when he started looking through his microscope at pond water. In a letter to Oldenburg, he described what he saw:

. . . there were many small green globules. Among these there were, besides, very many little animalcules, some were roundish, while others, a bit bigger, consisted of an oval (*2*).

van Leeuwenhoek described single-cell protozoa that probably included *Giardia*, judging from his sketches, making him the first man to ever see creatures that small. Some questioned such a claim. In 1680, the Royal Society sent a team to Delft and fully corroborated van Leeuwenhoek’s observations. Modern-day scientists have confirmed van Leeuwenhoek’s findings, even using some of his surviving microscopes (*3*).

In September 1683, van Leeuwenhoek reported to the Royal Society what he had seen in plaque from a healthy person’s mouth:

With great wonder, that, in the said matter there were many very little living animalcules very prettily a-moving. The biggest sort had the shape of [a rod]: these had a very strong and swift motion and shot through the water like a pike does through water. These were most always few in number. The second sort had the shape of [a small rod]. These often spun round like a top, and every now and then took a [spiral] course and were far more in number (*3*).

These words are the first known descriptions of bacteria. For 40 more years, he continued his correspondence with the Royal Society that published his letters, making him well known in Europe.

van Leeuwenhoek’s simple microscopes produced wondrous images but were exceedingly difficult to use. The instrument was ≈3 inches (7.5 cm) long, consisting of 2 metal plates, a lens placed in a hole made in the plates, and, on one side, a small metal pointer on which the specimen was held. The user would look through the opposite side of the device with a light source behind the specimen. The focal length of the lens was so short that the user needed to place an eye so close to the lens that the eyeball was nearly touching it. van Leeuwenhoek also kept his methods of producing the lenses secret, never teaching them to anyone. He had a businessman’s mind and wanted to keep trade secrets on his microscope’s lens formation and use. As he wrote to the Royal Society:

My method for seeing the very smallest animalcules and minute eels, I do not impart to others; nor how to see very many animalcules at one time. That I keep for myself alone (*3*).

Whereas a new world of microorganisms had been revealed, van Leeuwenhoek did not make any connection between the microorganisms and disease. The bacteria that van Leeuwenhoek observed were in humans who were not ill. No one else in the scientific and medical communities of the time made any connection to microorganisms as pathogens either. Until the seat of disease was reassessed from a change in the balance of humors to our modern medical pathophysiologic approach, the existence of such microorganisms was only a curiosity of nature (*4*). 

Despite van Leeuwenhoek’s technical advance, the cumbersome instruments he created were difficult to use. van Leeuwenhoek’s secrecy in fashioning lenses added to the limited use of microscopes for more than a century. Simple microscopes would eventually yield to compound microscopes when technical problems such as chromatic aberration were solved in the 19th Century. However, the limited interest in microscopy in the 17th and 18th Centuries cannot diminish van Leeuwenhoek’s pioneering contributions that fundamentally changed the way we think of the living world.
